# Single neuron transcriptomics identify SRSF/SR protein B52 as a regulator of axon growth and *Choline acetyltransferase* splicing

**DOI:** 10.1038/srep34952

**Published:** 2016-10-11

**Authors:** Boyin Liu, Torsten Bossing

**Affiliations:** 1 School of Biological Sciences, Bangor University, Deiniol Road, Bangor LL57 2UW, U.K; 2 School of Biomedical and Healthcare Sciences, Plymouth University, John Bull Building, Plymouth, PL6 8BU, U.K.

## Abstract

We removed single identified neurons from living *Drosophila* embryos to gain insight into the transcriptional control of developing neuronal networks. The microarray analysis of the transcriptome of two sibling neurons revealed seven differentially expressed transcripts between both neurons (threshold: log_2_1.4). One transcript encodes the RNA splicing factor B52. Loss of B52 increases growth of axon branches. B52 function is also required for *Choline acetyltransferase (ChAT* ) splicing. At the end of embryogenesis, loss of B52 function impedes splicing of ChAT, reduces acetylcholine synthesis, and extends the period of uncoordinated muscle twitches during larval hatching. ChAT regulation by SRSF proteins may be a conserved feature since changes in SRSF5 expression and increased acetylcholine levels in brains of bipolar disease patients have been reported recently.

One of the most complicated puzzles in brain development is how genes direct the formation of neuronal circuits that control a multitude of behaviours ranging from simple locomotion to complex social interactions. The fruit fly *Drosophila* is one of the most widely used neurogenetic models^1^,^2^. The systematic mapping of the stereotyped location and morphology of neurons and glial cells has made *Drosophila* one of the few organisms in which most of the components of the central nervous system (CNS) have been described^3^,^4^,^5^,^6^. The knowledge about the CNS building blocks combined with the ease and wealth of genetic manipulations available in *Drosophila* has accelerated our understanding of how genes determine cell fate, the pattern of division of neural stem cells, how axons navigate towards their target, and how synapses are formed and maintained^7^,^8^,^9^. The output of neural computation in the CNS directs the behaviour of an organism. *Drosophila* also has a long legacy in behavioural studies encompassing sexual behaviour, memory, circadian rhythms, taste, aggression, addiction among many others^10^,^11^. In the last few years *Drosophila* neurobiology has started to combine cellular with behavioural studies and to set out to identify all components and their connections making up neural circuits. The identification of each circuit becomes feasible because of the advent of public repositories of large sets of transgenic *Drosophila* stocks, which allows genetic manipulation and labelling of small subsets of identified neurons^3^. In addition, the invention of new tools allows limiting the expression in these subsets even further down to single cells^12^. Together, these tools allow the 3D construction by immunofluorescence and electron microscopy of neuronal circuits and the targeted activation, recording and silencing of neuronal subsets in a circuit^13^,^14^,^15^. Yet, to test the function of each connection and to predict the outcome of circuit activation, more information is needed about how each axon recognises its target, how an initial contact is stabilised and maintained, and finally which neurotransmitters and receptors are present at the newly formed synapse. Most of this information can be obtained by analysing the transcriptome of single neurons over time.

The availability of single cell driver lines enables cell purification using FACS (fluorescent activated cell sorting) or MACS (magnetic activated cell sorting) alone or in combination as a convenient method to enrich for specific cell types. Yet, these purifications often suffer from incomplete cell dissociation and/or the potential danger of transcriptional changes caused by transient cell culture^16^,^17^,^18^,^19^,^20^. To avoid cell dissociation, methods of tagging newly synthesized or translated RNA, such as PABP, TRAP or TU tagging, have been developed^21^,^22^,^23^. These methods allow RNA purification from lysed tissue but like MACS and FACS it is difficult to estimate the level of purity. Most recently, the isolation of nuclei tagged in specific cell types (INTACT) using a genetic tag has been designed to analyse the transcriptome of identified cells^24^. To be useful for single cell transcriptomics all these methods depend on the availability of transgenic animals, which only label a single cell type. In addition both methods often collect material from different animals capturing differences in transcription, which may be caused by differences in genetic background or nutrition and not by cell type. We recently developed a method, which allows the analysis of the transcriptome of single cells taken directly from living *Drosophila* embryos on whole genome microarrays^25^. Since cells are removed with a microcapillary, single cell labelling is not required and more than one cell can be removed and compared separately from the same embryo avoiding non cell-type specific differences.

Here we use our established method to identify transcriptional changes in two well-characterised embryonic *Drosophila* interneurons, vMP2 and dMP2, at the time when neural networks become functional^26^. We selected one of the identified transcripts, B52, which encodes a member of the Serine-rich splicing factor (SRSF) family, for functional analysis. Although B52 is widely expressed in the CNS including MP2 neurons, transcript expression appears higher in dMP2 than in vMP2. We were unable to detect any morphological or behavourial phenotype for the neural gain of B52 expression. Neural depletion of B52 function in the embryo increased the growth of the posterior axonal branch of the dMP2 neuron, interfered with the splicing of *ChAT pre-mRNA* and decreased the synthesis of ChAT. Reduced acetylcholine synthesis is most likely the cause for the delayed maturation in larval locomotion and the delay in hatching observed in B52 depleted animals. In summary, our experiments show that the analysis of the transcriptome of single neurons directly harvested from live brains is a powerful tool to identify transcripts with important functions in neuronal circuit formation.

## Results

### Transcriptional analysis of two single sibling neurons during neuronal network formation

**Figure 1:**
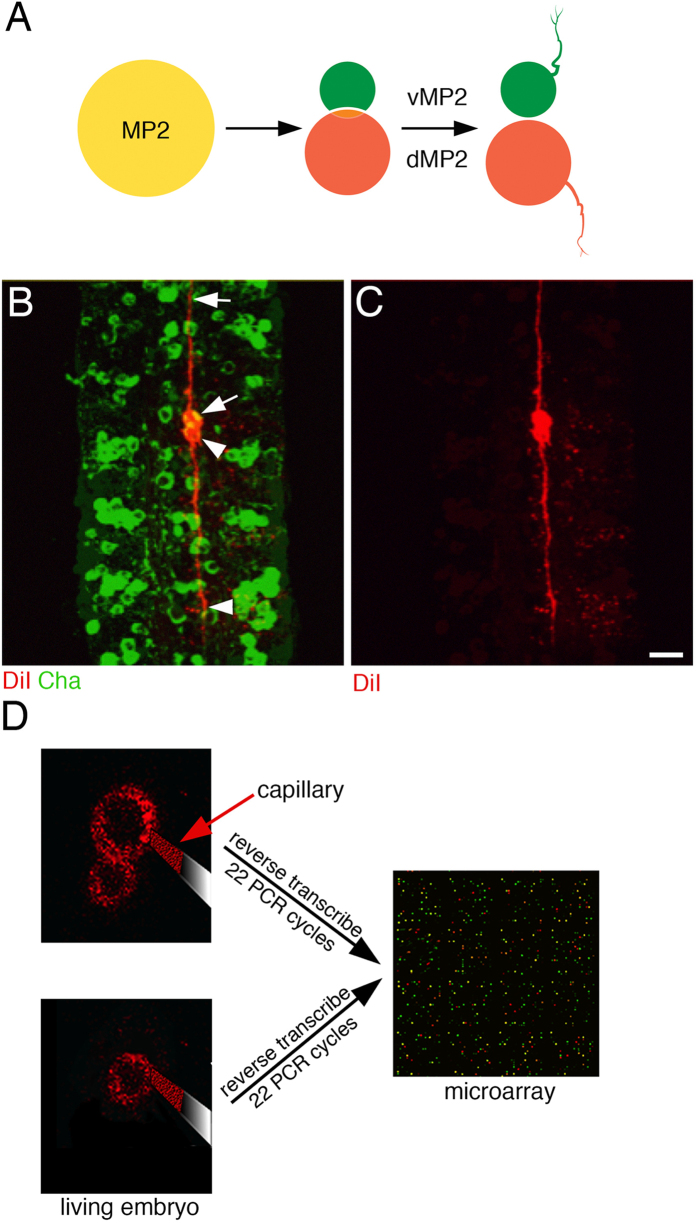
Transcriptional analysis of MP2 neurons. (**A**) The MP2 neural precursor divides only once to give rise to a vMP2 and a dMP2 interneuron. Both sibling neurons extend axons in opposite directions. (**B**,**C**) A MP2 precursor was labelled with the fluorescent dye DiI (red) in embryos expressing membrane bound green fluorescent protein (GFP) in cholinergic neurons (*ChAT-GAL4*/*UAS-CD8-GFP*). Embryos were allowed to develop for 17h after egg lay (ael). At this stage, vMP2 (arrow) has differentiated into an anterior projecting (arrow), cholinergic and dMP2 (arrowhead) into a posterior (arrowhead) projecting interneuron. C shows red channel only. (**D**) At 17 h ael both sibling neurons were separately removed with a capillary under epifluorescence and their transcriptomes were reverse transcribed, amplified and compared on whole genome microarrays. Horizontal views, Anterior is up; Scale bar: 10 μm (**B,C**).

We set out to test if the removal of single neurons from living *Drosophila* embryos and the subsequent analysis of their transcriptome would yield new insights into the formation of neuronal communication. To facilitate the functional analysis of identified transcripts we focused on the two MP2 sibling neurons, since these are a longstanding model system for neuronal development and offer readily available immunohistochemical as well as transgenic tools^27^,^28^. The simplest neuroblast lineage in the *Drosophila* CNS arises from the MP2 neural precursor (Fig. 1A). The precursor divides asymmetrically to give rise to a smaller, ventrally located neuron (vMP2), which projects anteriorly and a bigger, dorsally located neuron (dMP2), which projects posteriorly^5^,^27^. vMP2 s differentiate into cholinergic interneurons (Fig. 1B) whereas most thoracic and abdominal dMP2 neurons differentiate into projecting interneurons, which will die shortly before larval hatching. Immunohistochemistry did not detect ChAT, vGlut, Serotonin, Dopamine or GABA expression in dMP2 neurons (data not shown). dMP2 neurons located in posterior abdominal segments (A6–A9) survive, innervate hind- and midgut, and express and secrete insulin-like peptide 7^29^. We removed vMP2 and dMP2 neurons from the first abdominal segment (A1) of living embryos at 17 h of embryogenesis (Fig. 1D). Cell death of dMP2 neurons has first been reported at 19 h of embryogenesis^29^. Towards the end of embryogenesis (17 h after egg lay) action potentials can first be recorded from *Drosophila* neurons indicating the initiation of functional neuronal networks^26^. Using our established protocol^25^, four single MP2 siblings were removed from two different embryos (one sibling pair/embryo), the RNA was reverse transcribed and after amplification analysed on whole genome microarrays (FlyChip). Using stringent conditions, at 17 h MP2 sibling neurons show a very low number of differentially expressed transcripts (Table 1).

**Figure 2:**
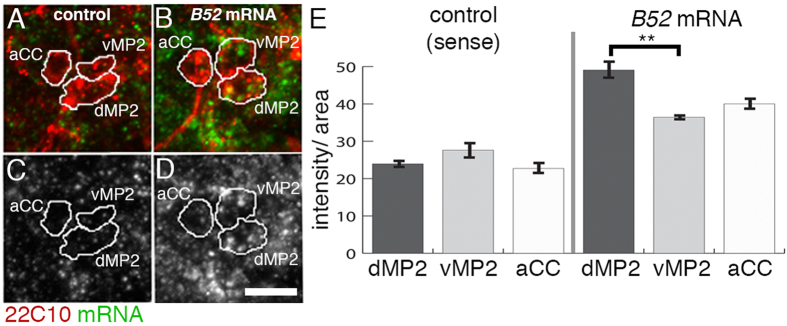
*B52* mRNA is expressed higher in dMP2 than vMP2 or aCC. (**A**–**D**) Panels show *in situ* hybridisation using sense mRNA as negative control (control, green) or anti-sense mRNA (B52 mRNA, green) to detect *B52* endogenous mRNA. vMP2, dMP2 interneurons and aCC motor neuron are detected by 22C10 antigen expression (red) and cells are outlined. (**C**,**D**) shows *in situ* signal only. Anterior is up. Scale, 5 μm (**E**) Quantification of *in situ* signal in vMP2, dMP2 and aCC. Sense signal is equal in all three cells but anti-sense signal detecting B52 mRNA is significantly stronger (p < 0.01, t-test) in dMP2 than vMP2. Bars are standard error. For each condition, sense and antisense, and cell type, 10 cells in three different embryos were measured.

To validate our study, we focused on the analysis of B52, which belongs to the evolutionary conserved SRSF protein family of pre-mRNA splicing factors including the B52 orthologues SRSF4 and 5, which are expressed in the human brain^30^,^31^. We used *in situ* hybridisation to detect B52 mRNA. In the CNS, B52 is widely expressed throughout embryogenesis (Fig. 2, Supplementary Fig. S1). Using 22C10/MAP1B as a marker for MP2 interneurons and aCC motor neurons^32^, we observed B52 relative mRNA amounts significantly higher in dMP2 than in vMP2 interneurons or aCC motor neurons (Fig. 2, Supplementary Fig. S1). We conclude that both, our microarray analysis and *in situ* RNA detection, support differential expression of B52 mRNA in MP2 neurons.

### Loss of B52 delays larval hatching without interfering with neuronal determination or gross axonal guidance

**Figure 3:**
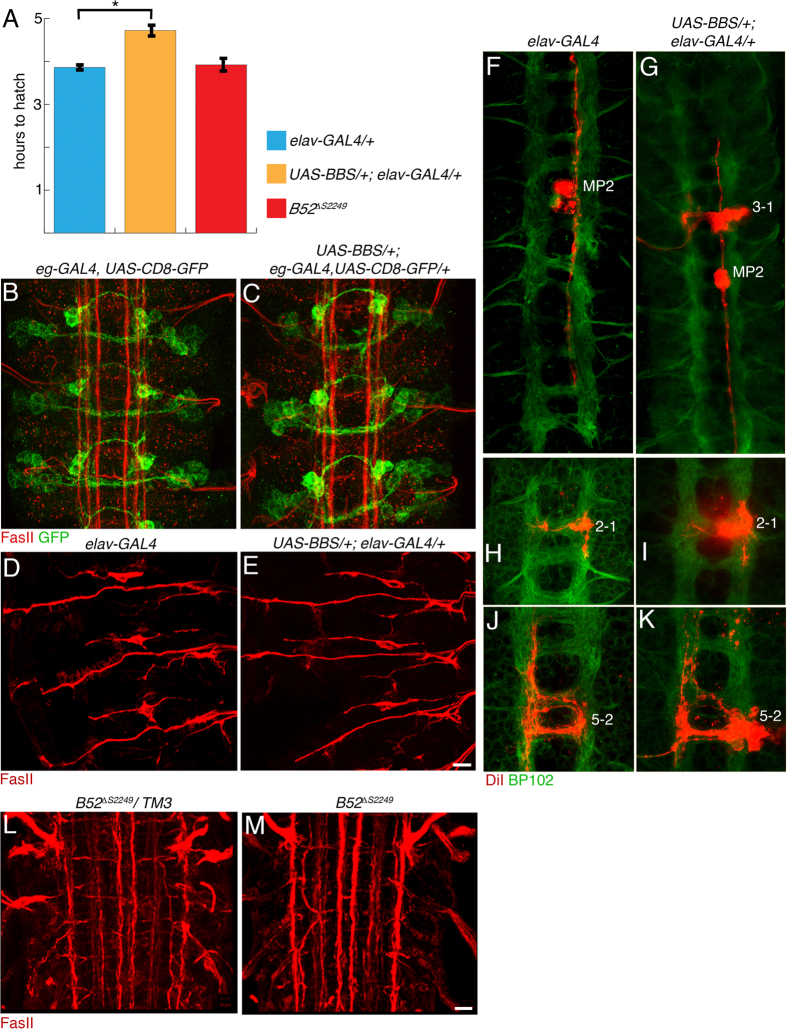
Loss of B52 function delays larval hatching, but does not interfere with determination, cell division and axon guidance. The genotypes are indicated at the top of each figure: *elav-GAL4* and, *B52*^*ΔS2249*^/*TM3,* control; *UAS-BBS*/+*; elav-GAL4*/+, B52 depletion; *eag-GAL4, UAS-CD8-GFP*, control; *UAS-BBS*/+*; eag-GAL4, UAS-CD8-GFP*/+, B52 depletion; *B52*^*ΔS2249*^, B52 null mutant. (**A**) Time for larval hatching for *elav-GAL4* (n = 38), *UAS-BBS*/+*; elav-GAL4*/+ (n = 37) and homozygous *B52*^*ΔS2249*^ mutants (n = 21) was measured starting at tracheal filling. Depletion of B52 function delays hatching by nearly 1 h (p = 0.02, t-test). *B52*^*ΔS2249*^ mutant embryos show no hatching delay. Error bars represent standard error. (**B**,**C**) Loss of B52 does not interfere with neuronal determination and differentiation. Neural precursors 2-4, 3-3, 6-4, 7-3 and their progeny are labelled by the expression of membrane-bound GFP (green*, eg-GAL4*/*UAS-myr-GFP*). At embryonic stage 17, no differences in cell number, differentiation and axonal projection between wild type and depletion of B52 function can be observed. (**D**,**E**) At stage 17, motorneurons were labelled with Fasciclin II antisera (red). Depletion of B52 does not affect muscle innervation. (**F**–**K**) Single neural precursors were labelled with the fluorescent dye, DiI (red), and their development followed *in vivo*. At stage 17, embryos were fixed, immunostained and the clones were recorded by confocal microscopy. Clones derived from precursor MP2, 3-1, 2-1 and 5-2 are shown. Depletion of B52 function does not interfere with neuronal determination, cell division or axon guidance. No changes in the axon scaffold (green, BP102) can be detected. (**L**,**M**) Axon projection pattern (red, FasII) in the CNS of 36 h post-hatching B52 homozygous mutant larvae are indistinguishable from wild type. Horizontal views, Anterior is up. Scale: 10 μm.

To overcome maternally deposited B52 protein^33^, we decided to interfere with B52 function by expressing a B52 binding site aptamer (*UAS-BBS*/+*; elav-GAL4*) in all embryonic neurons and neuroblasts. The aptamer delivers 60 B52 binding sites (BBS) arranged as a dodecamer of self-folding RNA molecules with five B52 optimised binding sites each. Expression of BBS has been shown to bind B52 thereby acting as B52 functional antagonist^34^. Depletion of B52 function in the embryonic CNS significantly delays larval hatching by nearly 1 h (25 ^o^C, Fig. 3A). Using tracheal filling as a start point, control embryos (*elav-GAL4*) take 3.86 h (+/−0.1 h, n = 38), whereas embryos with reduced B52 function take on average 4.76 h (+/−0.36 h, n = 37, p = 0.02). Since B52 mRNA is expressed throughout embryogenesis, a delay in hatching may indicate changes in neural stem cell determination, cell division or axonal guidance. To test for these changes, we depleted a small subset of neural stem cells and their progeny of B52 function (*UAS-BBS*/+*; eag-GAL4,UAS-CD8-GFP*). We were not able to detect any changes in cell morphology and cell number in the B52 depleted lineages (Fig. 3B,C). Depletion of B52 function also does not interfere with the guidance of motorneuronal axons and endplates (anti-Fasciclin II; Fig. 3D,E). Finally we labelled single neural stem cells with the fluorescent dye DiI^35^ to follow cell division and cell differentiation in embryos with (n = 32) and without (n = 22) B52 depleted function. We observed no changes in cell determination or cell division (Fig. 3F–K).

It is possible that interference with B52 function via expression of BBS does not sufficiently decrease B52 activity, explaining the lack of gross morphological phenotypes. Therefore we generated a B52 null mutant by imprecise P-element excision (see Material and Methods for characterization of mutant). *B52*^*∆S2249*^ mutant embryos do not show any hatching delay (Fig. 3A) but show retarded larval growth (Supplementary Movie S1), run out of B52 mRNA around 36 h (see below) and die around 40 h after larval hatching. The CNS of 36 h old *B52*^*∆S2249*^ larvae are 28% narrower than their heterozygous siblings or wild type embryos (n = 5, for both genotypes) but mutant CNSs show no obvious changes in their axonal scaffold (anti-Fasciclin II; Fig. 3L,M).

In summary, depletion of B52 function in the embryo seems not to interfere with neural stem cell determination, cell division or axonal guidance but causes a delay in larval hatching.

### Loss of B52 increases axonal growth

**Figure 4:**
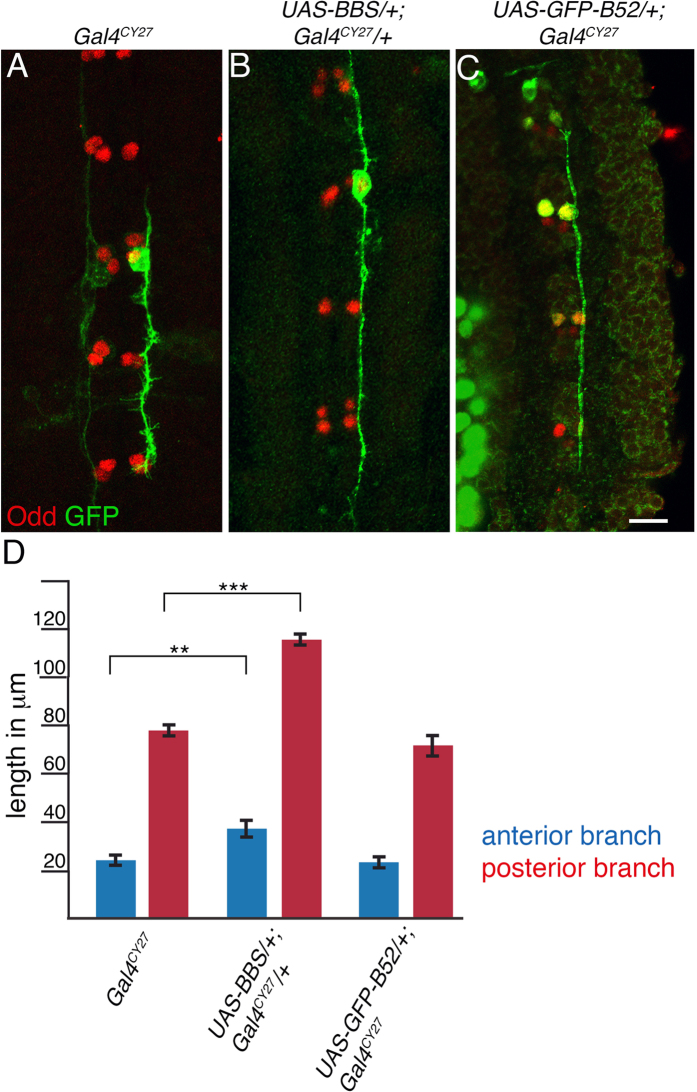
Loss of B52 increases axon extension. Genotypes indicated at the top of each picture are: *GAL4*^*CY27*^, control; *UAS-BBS*/+; *GAL4*^*CY27*^/+, B52 depletion; *GAL4*^*CY27*^/*UAS-GFP-B52*, gain of B52. Odd; Odd-skipped, transcription factor labelling MP1 and dMP2 neurons. (**A**–**C**) Mosaic clones were generated by injection of UAS-myr-GFP (green) plasmids into syncytial blastoderm embryos. (**B**) Only depletion of B52 function increases the length of the posterior branch of the dMP2 neuron (p = 0.002, t-test). (**C**) Expression of GFP-B52 (green) does not increase the length of axonal branches. GFP-B52 accumulates in the nucleus of dMP2 resulting in an overlap with the nuclear transcription factor Odd (red). Outline and axons of dMP2 are labelled by expression of the membrane bound myr-GFP. Horizontal views, Anterior is up. Scale, 10 μm. (**D**) Depletion but not gain of B52 function affects axon extension. N = 9 for all genotypes. Error bar indicates standard error.

To analyse on a single neuron level the phenotype caused by depletion of B52 function, we turned our focus back to dMP2 neurons. Labelling of dMP2s was performed by generating mosaic clones expressing myr-GFP^36^ into *GAL4*^*CY27*^ strain. *GAL4*^*CY27*^ drives expression strongly in dMP2 neurons, and transiently in vMP2 neurons plus a subset of late delaminating neuroblasts. Depletion of B52 function (*UAS-BBS*/+*; Gal4CY27*/+) increased anterior branch length by nearly 52% and posterior branch length by 46% (Fig. 4). In contrast, increased expression of full length B52 (*UAS-GFP-B52*/+*; Gal4*^*Cy27*^/+) did not interfere with growth of dMP2 axons branches (Fig. 4). We conclude that reduction of B52 function in dMP2 increases axonal growth.

### Loss of B52 interferes with maturation of larval movements, splicing of pre-mRNA and protein synthesis of ChAT

**Figure 5:**
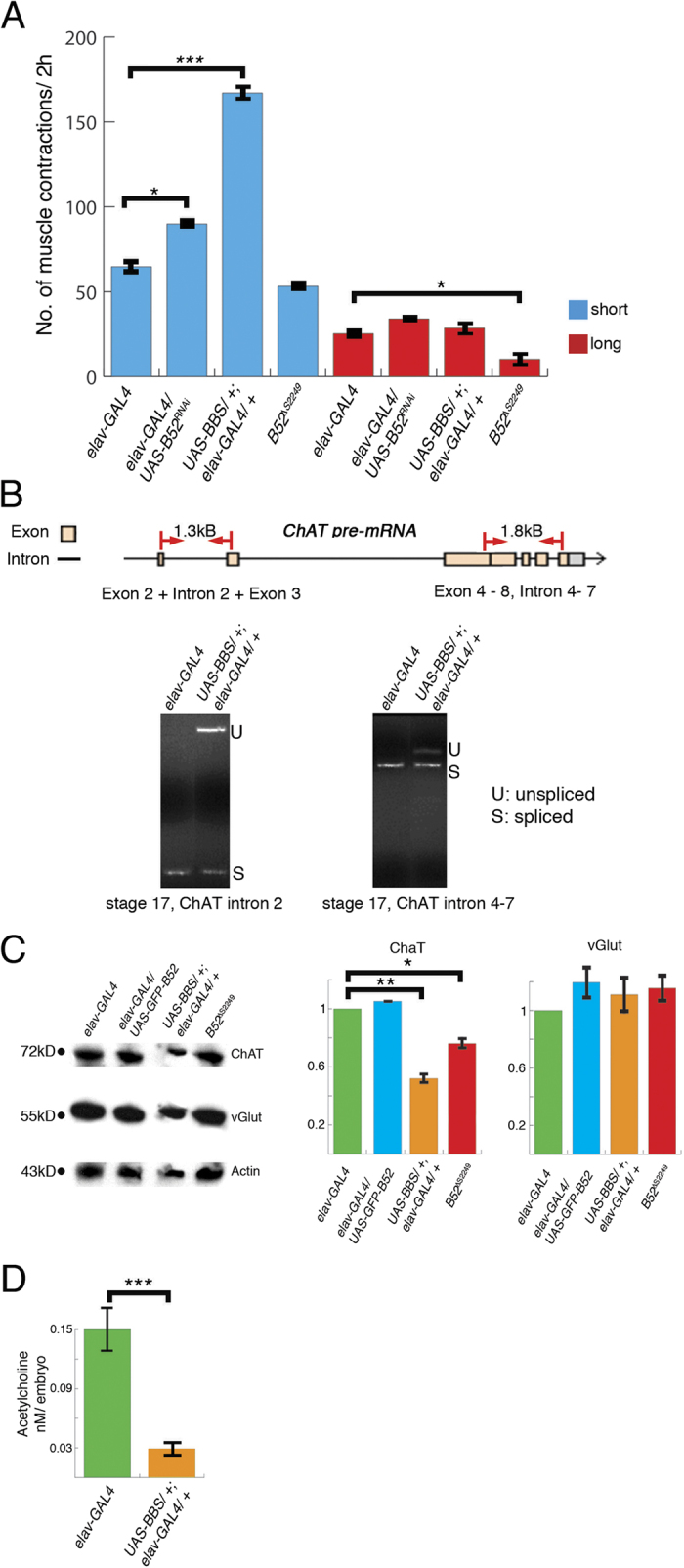
Depletion of B52 function affects larval hatching and Choline acteyltransferase (ChAT) splicing and synthesis. Genotypes are indicated at the top or bottom of each panel: *elav-GAL4*, control; *UAS-BBS*/+*; elav-GAL4*/+ and *elav-GAL4*/*UAS-B52*^*RNAi*^, B52 depletion; *elav-GAL4*/*UAS-GFP-B52*, gain of B52; *B52*^*ΔS2249*^, B52 homozygous mutant. (**A**) Compared to *elav-GAL4* larvae, larvae depleted of B52 function, either by expression of RNAi (p = 0.011) or BBS (p < 0.001, t-test), show an increase in short muscle contractions, short twitches (blue) which last up to 10 sec and often occur unilaterally in each segment. Depletion of B52 function does not alter the frequency of long bilateral muscle contractions (red), which last longer than 10 sec. Homozygous *B52*^*ΔS2249*^ mutants show a slight but not significant decrease in short contractions and a significant decrease in long contractions (p = 0.022, t-test). Bars represent standard error. N = 5 for all genotypes. (**B**) Depletion of B52 function (*UAS-BBS*/+*, elav-GAL4*/+) interferes with splicing of *ChAT pre-mRNA* in late embryos. Top: Schematic drawing of the *ChAT* gene structure in the examined region. Red lines mark position of primers. Bottom: Compared to controls (*elav-GAL4*), B52 depletion reduces splicing efficiency of *ChAT* intron 2 and intron 4–7. kB, kilobases; s, spliced; u, unspliced. (**C**) Compared to *elav-GAL4*, ChAT protein is significantly reduced in *UAS-BBS*/+*, elav-GAL4*/+ (p = 0.0015) and slightly reduced in *B52*^*Δ2249*^ mutants (p = 0.047, t-test). Western blot of nerve cords removed from late stage 17 embryos. VGlut expression does not change in gain or loss of B52. Graphs indicate relative band intensity of Western blot. Actin was used as loading control. Band intensity was adjusted to loading controls and normalized to elav-GAL4 control. Images are cropped. For uncut blots see Supplementary Fig. S4. (D) Depletion of B52 function (*UAS-BBS*/+*, elav-GAL4*/+) reduces Acetylcholine production. Graph presents average Acetylcholine concentration per embryo in stage 17 control (*elav-GAL4*) and B52 depleted embryos (*UAS-BBS*/+*, elav-GAL4*/+). Bars represent standard error (n = 2 experiments, 16 embryos/experiment, p < 0.001, t-test).

To obtain insight into the causes of hatching delays as a result of depletion of B52 function, we recorded larval movements and analysed the muscle contractions during a 2 h period starting from tracheal filling following neural expression of B52-RNAi or BBS. In wild type larvae movements first undergo a phase of uncoordinated muscle twitches lasting about 90 min until tracheal filling. After tracheal filling these short twitches are gradually replaced with coordinated waves of muscle contraction^37^. We defined short twitches as muscle contractions lasting up to 10 sec, occuring only unilaterally and often only in one segment. In contrast, long contractions last longer than 10 sec, pass through more than one segment, are bilateral and often include more than one peristaltic wave passing through the larvae. Compared to *elav-GAL4*, (Supplementary Movie S2) we observed in embryos where B52 function is reduced by neural expression of B52-RNAi (*elav-GAL4*/*UAS-B52*^*RNAi*^) *a* slight hatching delay and we find a significant increase in the number of short twitches (p = 0.011, Fig. 5A). Only a slight hatching delay upon B52-RNAi expression is not surprising as maternal B52 protein is still abundant at this age. In contrast, embryos in which B52 function is depleted by BBS expression (*UAS-BBS*/+*; elav-GAL4*/+) show delayed hatching and a threefold increase in the frequency of short twitches (p < 0.001; Fig. 5A, Supplementary Movie S3). No differences in the number of long contractions were observed (Fig. 5A). We conclude that B52 functional depletion delays the maturation of larval movement by extending the phase of uncoordinated twitching beyond tracheal filling.

Cholinergic interneurons are the major excitatory input for motorneurons, and control the development of the two phases of larval movement, twitching and long contractions^38^,^39^. Inhibitory GAB Aergic interneurons seem not to be involved in this process^40^. Since one of the main functions of B52 is pre-mRNA splicing^41^ we sought to investigate if B52 may also control the splicing of *Choline-acetyltransferase (ChAT*), which *encodes* the enzyme involved in the last step of the biosynthesis of the neurotransmitter acetylcholine. We focused on the splicing of intron 2 and introns 4–7 of *ChAT pre-mRNA*. Indeed, a semi-quantitative analysis by RT-PCR of RNA extracted from 19 h old embryonic CNS (stage 17) shows that neural depletion of B52 function (*UAS-BBS*/+*; elav-GAL4*/+) reduces splicing of intron 2 and introns 4–7 of ChAT (Fig. 5B). This lack of efficient splicing leads to a reduction in ChAT protein synthesis (Fig. 5C). We also tested if the reduction of ChAT synthesis would lower acetylcholine synthesis. We discovered that acetylcholine synthesis is reduced by 80% in embryos after neural depletion of B52 function (Fig. 5D).

There is only a slight reduction of ChAT protein in B52 homozygous mutants (Fig. 5C), which do not show any hatching delay and only run out of B52 transcript 36 h after larval hatching.

**Figure 6:**
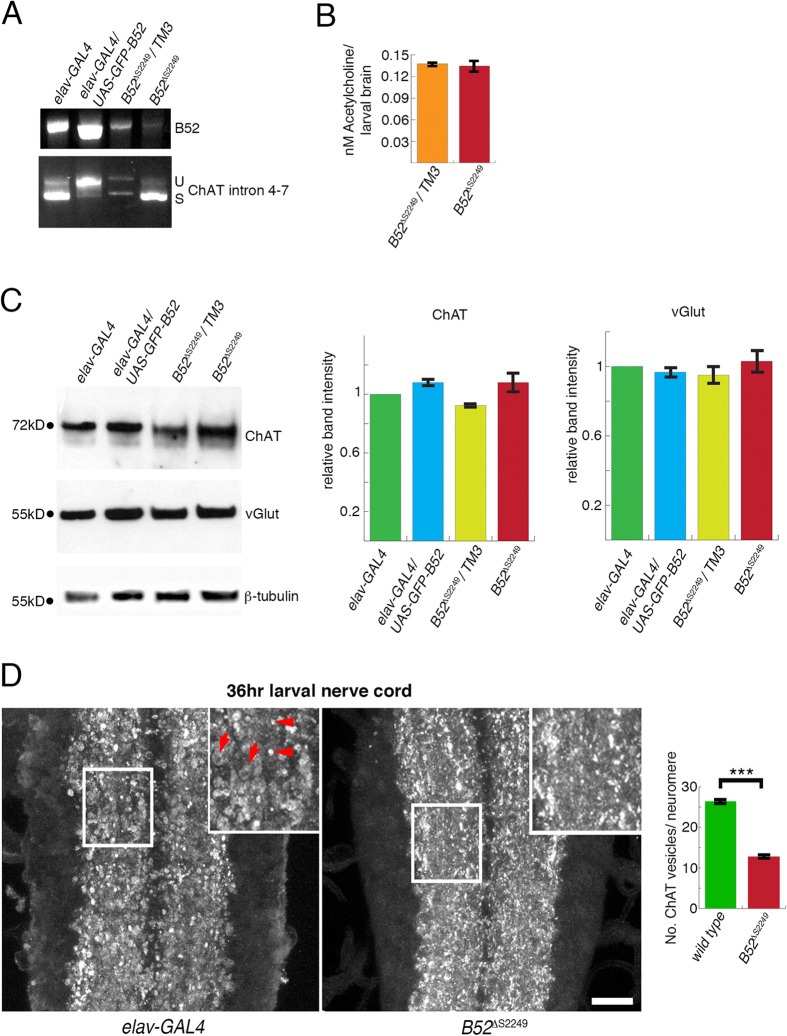
Gain but not loss of B52 affects splicing of *ChAT.* Genotypes are indicated at the top or bottom of each panel: *elav-GAL4 and B52*^*ΔS2249*^/*TM3*, control; *elav-GAL4*/*UAS-GFP-B52*, gain of B52; *UAS-BBS*/+*; elav-GAL4*/+, B52 depletion; *B52*^*ΔS2249*^, B52 homozygous mutant. (**A**) Top panel: In 36 h larvae, increased neuronal expression of B52 (*elav-GAL4*/*UAS-GFP-B52*) raises the amount of B52 RNA and loss of B52 in heterozygous (*B52*^*Δ2249*^/*TM3*) and homozygous larvae (*B52*^*Δ2249*^) reduces B52 mRNA. In homozygous mutants B52 mRNA is barely detectable. Bottom panel: Gain of *B52* mRNA results in a reduced efficiency of splicing of Intron 4–7 of *ChAT*. Whereas loss of *B52* shows the opposite effect and splicing of intron 4–7 in *B52*^*Δ2249*^ mutant larvae seems as efficient as in controls (*elav-GAL4*). Images are cropped. For uncut images see Supplementary Fig. S4. (**B**) Loss of *B52* does not change Acetylcholine synthesis in larval brains. Average acetycholine level per larval brain is shown (n = 2 experiments, 10 larval brains/experiment, bars are standard error). (**C**) Neither gain nor loss of *B52* changes levels of ChAT protein (top) or vGlut levels (middle). Tubulin was used a loading control (bottom). Band intensity was adjusted to loading controls and normalized to elav-GAL4 control. Graphs represent band intensity for ChAT and vGlut as indicated on top of each graph. Three independent lysates were prepared, separated by SDS gelectrophoresis and blotted. Error bars show standard error. Images of blots are cropped. For uncut images see Supplementary Fig. S4. (**D**) Loss of B52 does not cause ectopic or increased expression of ChAT. ChAT in control larval CNS (left) and *B52* homozygous mutants (middle) can be mainly detected along the longitudinal axon tracts. Yet, in control embryos, ChAT is present in structures resembling neuronal cell bodies (arrow, inset) as well as in small vesicles (arrowhead). In mutants, ChAT is limited to vesicles. Insets are 1.6x magnifications of framed area. Graph (right) represents the number of ChAT positive vesicles/neuromere. Vesicles in 2^nd^ and 3^rd^ abdominal neuromere with a diameter above 1 μm were counted. Wild type: 26.43 vesicles per segment, n = 18; B52^*Δ*S2249^: 12.78 vesicles/neuromere, n = 14. p > 0.001; Bars represent standard error. Horizontal views, Anterior is up. Scale: 10 μm (16 μm in inset).

In 36 h post-hatching *B52*^*∆S2249*^ mutant larvae, shortly before they contract permanently, B52 RNA is barely detectable (Fig. 6A, top) but surprisingly introns 4–7 are spliced as efficiently as in control embryos (Fig. 6A, bottom). Loss of only one copy of B52 in heterozygotes (*B52*^*∆S2249*^/*TM3*^*GFP*^) reduces B52 mRNA (Fig. 6A, top) and interferes with splicing of introns 4–7, an effect which can also be seen in the increased neural expression of B52 (*elav-GAL4*/*UAS-GFP-B52*; Fig. 6A, bottom). B52 does not affect splicing of all introns in the ChAT pre-mRNA. The coding sequence (CDS) for the vesicular acetylcholine transporter (*vAChT*) is located inside the first intron of the *ChAT* CDS and originates from an alternative splicing of the *ChAT* CDS. *vAChT* has only one intron and splicing is not affected by gain or by loss of B52 (Supplementary Fig. S2).

The rigidity of the *B52*^*∆S2249*^ homozygous mutant 2^nd^ instar larvae indicates that hyper-contraction and not failure of contraction of muscles may be the cause for the observed paralysis (Supplementary Movie S3). We tested if immobility might be caused by changes in the interneuronal neurotransmitter acetylcholine or the motorneuronal neurotransmitter glutamate. First, we measured acetylcholine levels in 36 h larval nerve cords. We did not detect changes in acetylcholine levels between heterozygous and homozygous mutants (Fig. 6B). Second, we examined ChAT and vGlut protein levels at 36 h after larval hatching, ChAT protein synthesis is not significantly altered in homozygous *B52*^*∆S2249*^ mutants as compared to their heterozygous siblings or *elav-GAL4* larvae (Fig. 6C, top). Also, raising neural B52 levels (*elav-GAL4*/*UAS-GFP-B52*) does not affect ChAT synthesis (Fig. 6C). We used the vesicular Glutamate transporter (vGlut) as indicator for glutamate levels^42^. Gain or loss of B52 function does not significantly alter vGlut protein synthesis (Fig. 6C, middle). Although we detected no differences in ChAT protein levels our immunodetection in the CNS of 36 h old *B52*^*∆S2249*^ homozygous larvae and controls (*elav-GAL4*) reveals differences in ChAT localisation. In controls and mutants, ChAT accumulates along the longitudinal axon tracts. In controls, ChAT is present in bigger circular structures (arrow, Fig. 6D inset) and in smaller dots (arrowhead, Fig. 6D inset) but in mutants *B52*^*∆S2249*^ ChAT can mainly be detected in dots (Fig. 6D, Supplementary Fig. S3). We counted and measured the diameter of all ChAT positive vesicles in the 2^nd^ and 3^rd^ abdominal neuromeres (Fig. 6D). Compared to wild type (26.43 vesicles/segment; n = 18 segments), in *B52*^*∆S2249*^ mutants the number of vesicles of above 1 μm diameter is significantly reduced (12.78 vesicles/segment; n = 14 segments; p > 0.001). We detect no significant differences in the average diameter size between vesicles in wild type (2.05 μm) and *B52*^*∆S2249*^ (2.2 μm).

Together our data indicates that increased B52 levels affect splicing of ChAT in 2^nd^ instar larvae. In contrast to embryos with functionally depleted B52, B52 loss or gain in 2^nd^ instar larvae does not change ChAT protein expression or Acetylcholine levels.

## Discussion

We tested if the analysis of the transcriptome of single neurons removed from the ventral nerve cord of living brains can be used to identify new molecules directing neuronal network formation. We harvested single MP2 neurons at a time when axonal guidance draws to a close and the first evoked action potentials can be recorded^26^. We focused on MP2 neurons because of their common origin and their two very distinct fates, an anterior projecting cholinergic interneuron versus a posterior projecting interneuron destined for apoptosis. Our chosen time point of cell removal, stage 17, a time when neurons are nearly fully differentiated and when neuronal networks become functional, should increase the possibility of identifying transcripts involved in network function and to exclude transcripts controlling cell determination. Indeed, the transcripts we discovered are involved in cell contact, cellular maintenance and epigenetic control of chromatin states. Due to their different fates we expected a vast array of transcriptional differences between the two MP2 siblings, yet we only discovered 7 out of 2631 transcripts, which are differentially expressed (threshold 1.4x). This number of differentially expressed transcripts is dependent on the high stringency filters we applied and will also vary in the course of development. Yet, the lineage of MP2 neurons also sets them apart from all other interneurons in the lateral CNS because their precursor only divides once, generating neurons directly and not via a transient ganglion mother cell (gmc)^5^. Our preliminary results comparing the transcriptome of sibling interneurons from a neuroblast (Nb) following a conventional division pattern (Nb5-2) reveal a much higher diversity. In contrast to MP2, which divides only once, Nb5-2 can undergo up to 20 divisions, generating 20 gmc’s and 40 neurons. During these divisions a cascade of temporal factors controls changes in neuronal identity^43^. Applying the same stringent criteria as in our MP2 analysis, two interneurons removed from the same Nb5-2 lineage differ in 569 transcripts out of 1226 (46,2%; Bossing, unpublished results). Comparing interneurons from different Nbs (Nb6-1 vs Nb7-1) increases molecular diversity even further, 633 out of 1232 transcripts are differentially expressed (51.4%, Bossing, unpublished results). These preliminary results are in line with previous publications reporting a high molecular diversity of interneurons^44^,^45^. The low number of differential expressed transcripts between MP2 neurons seems to be the exception.

### The SRSF protein B52 regulates ChAT splicing

One transcript we found expressed higher in dMP2 than vMP2 encodes the SRSF protein B52, which is the closest *Drosophila* orthologue to human SRSF6. SRSF proteins were first characterised as pre-mRNA alternative and constitutive splicing factors^46^. In addition to their role in splicing, several SRSF proteins have been shown to regulate transcription elongation, RNA export, decay, translation^47^, and for *B52,* transcriptional regulation^48^. Interestingly, SRSF4, one of the human orthologues of B52 expressed in the CNS, has been shown to be involved in alternative splicing of Tau and the origin of Frontotemporal Dementia^31^,^49^. We studied the function of B52 in neural development in *Drosophila* in more detail. *B52* is maternally deposited and expressed throughout the CNS. Since we chose to analyse the transcriptome of MP2 siblings at the end of embryogenesis, we would not expect *B52* to be a crucial factor in cell fate determination. Indeed neural depletion of B52 function by pan-neural expression of a RNA aptamer or in subsets of neuroblasts and their progeny does not create gross morphological defects. Yet, we discovered that functional depletion of B52 affects axonal growth and the pre-mRNA splicing and biosynthesis of ChAT.

One of the B52 splicing targets identified by genomic systematic evolution of ligands by exponential enrichment (SELEX)^50^ and co-immunoprecipitation^51^ is the BTB/POZ transcription factor *longitudinal lacking, (lola*), which controls axon extension via the transcription of *spire*, an actin nucleation protein^52^. We show that interference with B52 function in dMP2 increases axonal growth. Whereas increased expression of B52 does not alter axonal growth suggesting a saturation of the B52 target in the wild type condition. Thus we speculate that B52 may control axonal growth via splicing of *lola* for which at least 25 different isoforms have been reported (www.flybase.org).

In contrast to *lola* pre-mRNA, *ChAT* pre-mRNA has not yet been identified as a B52 splicing target. Therefore we cannot exclude that the observed splicing regulation of *ChAT* pre-mRNA is indirectly mediated via a yet unknown B52 target. In the embryo, depletion of B52 function by the expression of *UAS-BBS* results in accumulation of unspliced *ChAT* pre-mRNA and consequently a reduction in ChAT protein synthesis and reduced acetylcholine synthesis. Cholinergic interneurons are the only excitatory input for embryonic motor neurons. During the transition from uncoordinated muscle twitches to coordinated peristaltic muscle contractions, the shape of motorneuronal dendrites is fine tuned by presynaptic contact as well as acetylcholine release. In *ChAT* mutants, larvae do not hatch and motorneuronal dendrites overgrow^39^. A reduction of ChAT synthesis and acetylcholine synthesis as seen upon embryonic B52 functional depletion may therefore cause the observed increase in uncoordinated movements and the hatching delay.

At the end of their life B52 homozygous mutant larvae are smaller than their heterozygous siblings and appear paralysed. Both phenotypes might be explained by deficient Ecdysone signalling. A reduction in larval size is not unexpected because the Ecdysone receptor (EcR)^50^ and many ecdysone inducible transcripts^51^ have been identified as B52 splicing targets. Larvae without EcR function do not moult, remain small and gradually become immobile and insensitive to touch^53^.

Pre-mRNA splicing depends on the stoichiometry of snRNPs and splice factors. In addition, phosphorylation of SRSF proteins defines their binding specificity, function and localisation^54^,^55^. The accumulation of unspliced *ChAT* pre-mRNA caused by the depletion of B52 function in embryos and by targeted expression of B52 in 2^nd^ instar larvae, two opposing manipulations, support that stoichiometry plays an important role for efficient splicing. Yet, changes in stoichiometry appear insufficient to explain the efficient splicing of Cha introns 4–7 in *B52*^*∆S2249*^ mutant larvae, which show a severe reduction of *B52* mRNA. The different splicing phenotypes between mutant and functionally depleted larvae may be due to partial redundancy between B52 and the second SRSF protein in *Drosophila*, Asf/SRSF2. Target specificity of B52 and Asf proteins shows some overlap^51^. Therefore the increase in artificial B52 binding sites with the expression of the BBS aptamer may not only titrate out B52 but also Asf leading to a functional depletion of both SRSF proteins. In contrast, in *B52* homozygous mutants, the loss of B52 protein can still be compensated by the presence of Asf protein.

Our work shows that functional depletion of B52 reduces *ChAT* pre-mRNA splicing and ChAT protein synthesis although the exact regulation of *ChAT* pre-mRNA by B52 awaits further investigation. The lower expression of B52 in the cholinergic vMP2 than in the non-cholinergic dMP2 may be a compromise allowing vMP2 to still splice *ChAT* pre-mRNA efficiently and continue axonal growth.

Bipolar disorder (BD) is a devastating mental disease, in which the patient fluctuates between depressive and maniac mood swings. Elevated acetylcholine levels are the trigger for the depressive phases^56^. A recent study tried to identify transcriptional changes in the dorsolateral prefrontal cortex in postmortem brains of patients who suffered from bipolar disorder. Only five differentially expressed genes and 12 differentially expressed transcripts were discovered^57^. Interestingly, one of the transcripts encodes for the SRSF5 protein, an orthologue of B52, which was significantly downregulated in BD patients. We show that depletion of B52 function decreases ChAT protein, and subsequently acetylcholine levels. Therefore, not only are ChAT and SRSF proteins widely conserved throughout the animal kingdom but also the mechanism controlling splicing of *ChAT* pre-mRNA by SRSF proteins might be conserved.

## Materials and Methods

### Acetylcholine Measurements

Measurements of Acetylcholine were performed using a commercially available kit (Abcam) and a slightly modified protocol. Of each genotype 16 embryos or 10 larval brains were transferred into 1.5 ml centrifuge tubes, spun at maximum speed in a table centrifuge at 4 ^o^C, shock frozen in liquid nitrogen and ground up frozen in 40 ul assay buffer with a pestle attached to an electronic screwdriver at RT for 3 min. The sample was frozen again at −20 ^o^C for 10 min. Frozen sample was ground again for 2 min until completely thawed. Finally, the sample was spun at maximum speed in a table centrifuge at 4 ^o^C for 2 min. To 6 ul supernatant or calibration standard 2 ul Enzyme Mix and for two lysates an additional 2 μl Acetylcholinesterase was added. Samples were incubated for 30 min in the dark at RT. Adsorption of calibration standards and lysates were measured on a Nanodrop at 570 nm and 600 nm.

### Fly stocks

We used the following fly stocks: *eag-GAL4*^58^, *elav-GAL4*^59^, *GAL4*^*CY27 *^60, *P*{*lacW} B52*(*s2249P*}/*TM3* (Bloomington 10265), *UAS-BBS*^34^; *UAS-GFP-B52*^51^; *UAS-mCD8-GFP*^61^; *UAS-myr-GFP*^62^, *UAS-B52*^*RNAi*^ (TRiP.HMS01661, Bloomington 111038), *UAS-B52*^*RNAi*^ (KK108253, VDRC v101740).

### Generation of B52 mutants

We generated the B52 mutant, *B52*^*Δs2249*^, by crossing a stable source of transposase (*Δ2-3sb*/*TM6b*) with the hypomorphic B52 allele, *P*{*lacW*} *B52*(*s2249P*}/*TM3*. F2 progeny were selected for white eyes and crossed to each other to test for lethality. The genomic DNA of lethal strains was tested for deletions by PCR using primers located at −967 Bp, 3’UTR of *Hrb87f*, and at +1728 Bp. Sequencing identified a deletion starting at −456 Bp and extending to +165 Bp, eliminating the transcriptional start and the first 55aa of the ORF. *B52*^*Δs2249*^ fails to complement *P*{*lacW*} *B52*(*s2249P*} and PCR analysis of larval cDNA confirms that the deletion does not affect the expression of the *Hrb87f* gene immediately upstream of *B52. B52*^*Δs2249*^ homozygous mutants die as 2^nd^ instar larvae around 40 h after larval hatching.

### Immunohistochemistry, Western Blots and generation of mosaic expression

The following antibodies were used: BP102 (mouse, 1:1000; kind gift from N. Patel), anti-ChAT (mouse, 1:50 for immuno, 1:500 for Western Blots; DSHB), anti-FasII (mouse, 1:20; DSHB), anti-Futsch (mouse, 1:20; DSHB), anti-GFP (rabbit, 1:1000; kind gift from U. Mayor), anti-GFP (mouse, 3E10, 1:50; Life Technologies), anti-Odd (rabbit, 1:1000; kind gift from Jim Skeath), anti-vGlut (rabbit, 1:1000 in Immuno and 1:10,000 in Western Blots; kind gift from H. Aberle). Secondary antibodies were coupled to Alexa 488, 568 (Life Technologies). Immunohistochemistry was performed as described in^63^. Embryos were embedded in 70% Glycerol/Vectashield (3/1).

Due to increased background using whole embryo lysates, we blotted only lysates of dissected late embryonic brains (stage 17). 8 embryonic or 5 larval brains were transferred into 1.5 ml centrifuge tubes and lysed for 2 min with a plastic pestle attached to an electronic screwdriver in a 200 ul mixture of HEPES buffer (0.32 M sucrose, 4 mM HEPES, 0.5% Complete Protease Inhibitor [Roche Applied], pH = 7.4) and Laemmli loading buffer. After lysis, samples were boiled for 5 min and stored at −20 ^o^C. Gel separation at 120 V, wet transfer at 30 mA overnight at 4 ^o^C, antibody incubation and luminescence detection (Super Signal West pico; Pierce) followed standard procedures. Band intensity was analysed using the Gel Analysis module in Image J.

Mosaic expression of myr-GFP was generated as described previously^36^. To reduce the frequency of cells expressing GFP we injected UAS-myr-GFP plasmids at a concentration of 200 ng/ul into CY27^GAL4^ embryos at 2.5 hours after egg lay (late syncytial blastoderm). Embryos were inspected under epifluorescence at stage 17 and subjected to immunohistochemistry^36^.

### *In situ* hybridisation

*In situ* hybridisation with *in vitro* transcribed B52 Digoxigenin labelled RNA has been described previously^60^. Immunohistochemistry was performed after completion of the hybridisation protocol. For signal amplification we used secondary antibodies coupled to alkaline phosphatase together with Fast Red (Sigma). To quantify RNA expression we collected and merged four Z sections (0.35 μm intervals) around the biggest diameter of each neuron. We used Photoshop CS6 to select the outline of each neuron and measure the area and integrated density (sum of all intensities). To adjust for different cell sizes we divided the integrated density/area. In total, we measured selected ten neurons of each type from thoracic and abdominal segments of three embryos.

### Single cell transcriptome and RT-PCR

The protocols for the generation of cDNA from single cells, microarray hybridisation and the statistical analysis of array results have been published in detail previously^25^.

To generate 1^st^ strand templates for the testing of splice forms, we dissected 10 embryonic and 5 larval CNSs from each genotype and harvested the RNA using the GenElute Mammalian Total RNA Kit (Sigma). Samples were analysed by reverse transcribing total RNA from larval CNSs using Superscript II (Clontech) and primers from the Smarter PCR cDNA synthesis kit (Clontech). We tested for the presence or absence of the splice product by amplification through 35 PCR cycles. For every PCR we used 100 ng of 1^st^ strand cDNA as templates. All RT PCRs were repeated twice using freshly dissected CNSs as RNA source. The primers used are: Cha Intron 2 forward CCAAAGAAATGGCTCTCAACG, Cha Intron 2 reverse CAGCAGATACTGATGCAGCCG, Cha Intron 4–7 forward GCAGGACTCGCAGTTCCTGCC, Cha Intron 4–7 reverse CGGATGCGGATTGTAGGAGCA, vAChT forward GGATGTCGTGCAAGTTGAGTGG, VAChT reverse GGAAATTGCTTAGCTCTCGC.

### Time lapse movies

2 h old embryos were bleached, aligned on agar slices, and transferred onto glue coated coverslips. Embryos were covered with 10 s Voltalef Oil and allowed to develop another 16 h at 25 ^o^C. 18 h old embryos were inspected under a dissecting microscope every 30 min for tracheal filling. After tracheal tubes were filled with air we started the recording. Embryos were recorded on an inverted Nikon Eclipse TE300 with 100x magnification using a Hamamatsu Orca 100 camera and HCImage as image capture software. During recording, embryos were kept in a dark room with the temperature set at 20 °C. The intensity of transmitted light projected onto the embryos was set at minimum level. Time interval between each capture was set at 5 second for a recording period of 2 hours or more.

## Additional Information

**How to cite this article**: Liu, B. and Bossing, T. Single neuron transcriptomics identify SRSF/SR protein B52 as a regulator of axon growth and *Choline acetyltransferase* splicing. *Sci. Rep.* **6**, 34952; doi: 10.1038/srep34952 (2016).

## Supplementary Material

Supplementary Movie S1

Supplementary Movie S2

Supplementary Movie S3

Supplementary Information

## Figures and Tables

**Table 1:** Differentially expressed transcripts detected in dMP2 versus vMP2 neurons.

Gene	*wrapper*	*CG31855*	*CG7433*	*B52*	*fis1*	*trx*	*DMAP1*
Fold Expression (log_2_FC dMP2 vs vMP2)	6.06	4.27	3.96	3.92	1.64	−2.84	−3.53
Function	Neurexin IV receptor	Ubiquitin ligase	Trans-aminase	Splicing Factor	Mitochondria	Chromatin binding	Chromatin binding

At 17 h in embryonic development, dMP2 and vMP2 neurons are highly similar. In two biological replicates, only 7 out of 2631 transcripts show changes in expression levels of more than 1.4 fold up or down, have a high spot quality of 8 or more and a p-value below 0.05. Log_2_FC: Logarithmic base 2 Fold Change in expression levels.
