# Supplementary material for: Single neuron transcriptomics identify SRSF/SR protein B52 as a regulator of axon growth and Choline acetyltransferase splicing
Source: Sci Rep. 2016 Oct 11;6:34952. doi: 10.1038/srep34952 (PMC5057162; doi:10.1038/srep34952)
Supplement: Supplementary Information [file srep34952-s4.pdf]

Single neuron transcriptomics identify SRSF/ SR protein B52 as a regulator of axon growth and *Choline acetyltransferase* splicing

Boyin Liu and Torsten Bossing

Supplementary Information

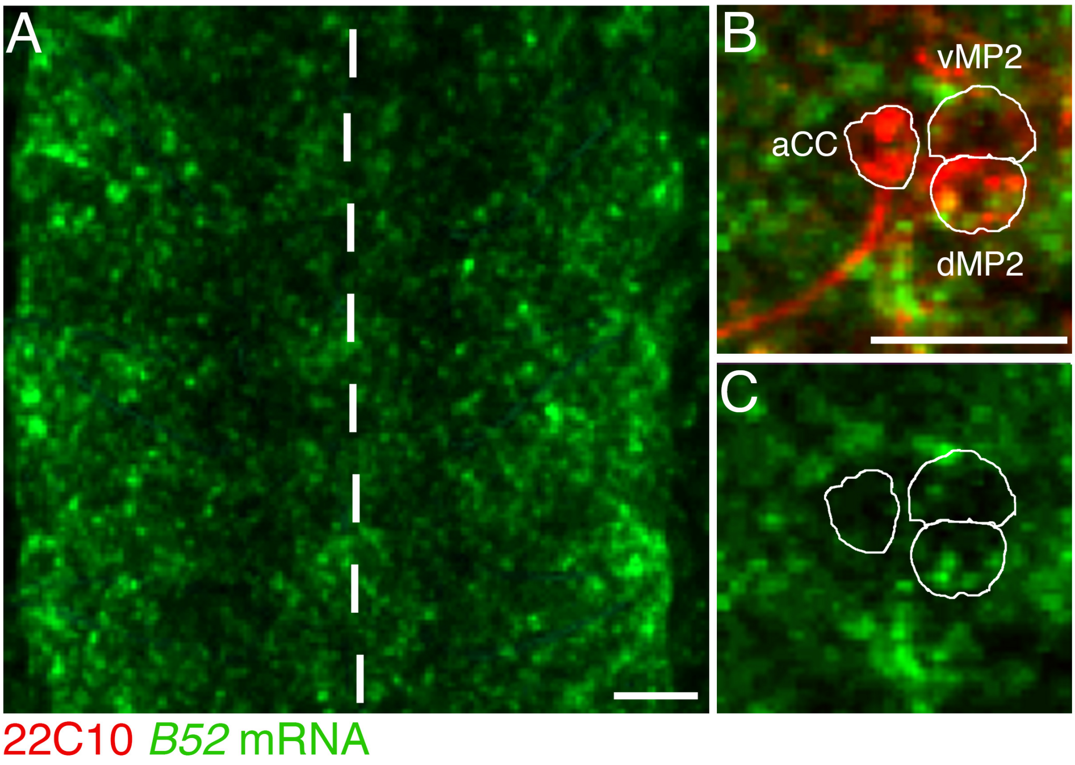

Figure S1 B52 is widely expressed in the embryonic CNS.

(A) B52 mRNA is widely but not uniformly expressed in the embryonic CNS at 17h after egg lay (ael). (B, C) Higher levels of B52 mRNA can be detected in dMP2 than in vMP2 or aCC motor neuron. Horizontal views, Anterior is up; Scale bar: 10μm

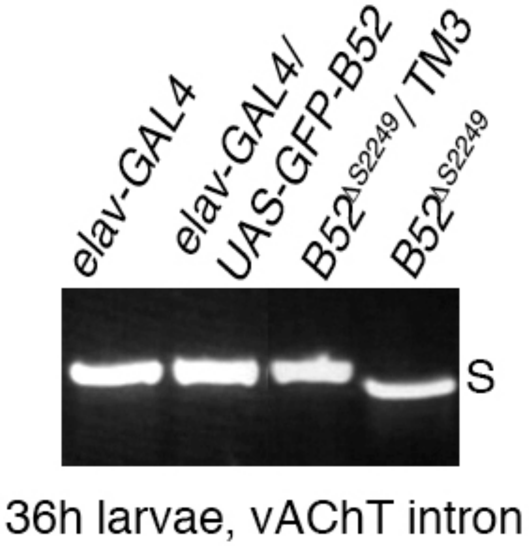

Figure S2 B52 gain or loss does not affect splicing of VAChT intron. Genotypes are indicated at the bottom of picture. *elav::GAL4,* control; *elav::GAL4/ UAS::GFP-B52,* Gain of B52; $B52^{\Delta S2249}$*/ TM3*; heterozygous mutant; $B52^{\Delta S2249}$*,* homozygous mutant . Samples were analysed by reverse transcribing total RNA from larval CNS and testing for the presence of the splice product by amplification through 35 PCR cycles. Equal amounts of 1st strand cDNA served as templates. Only spliced message will generate a product. S; spliced.

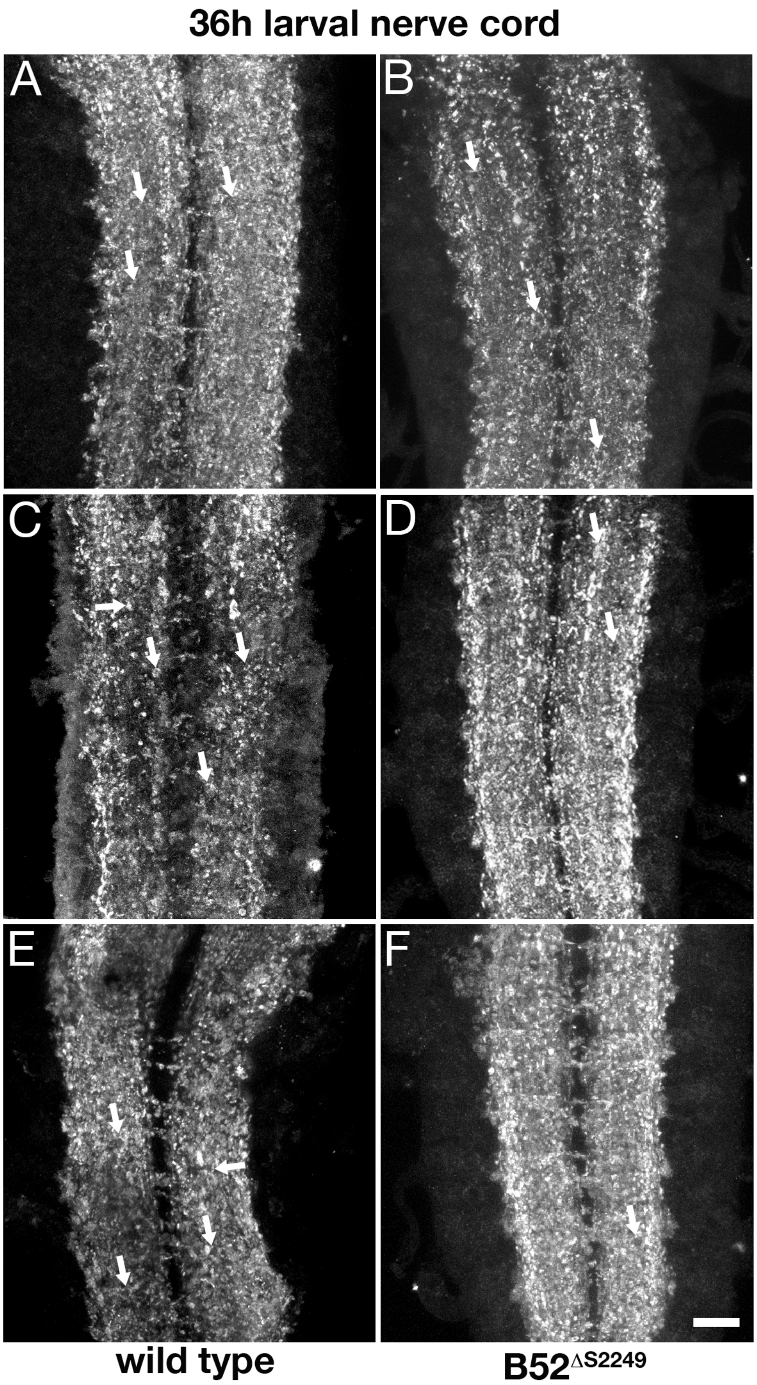

Figure S3 Loss of B52 changes localisation of ChAT in the neuropile. ChAT immunostaining in 36h larval nerve cords of wild type (left, A, C, E) and B52 mutants (right, B, D, F). In the wild type neuropile ChAT staining can be frequently detected in vesicle-like structures (arrows). In B52 mutants, the numbers of vesicles is reduced and ChAT can be predominantly detected in small dots. Scale bar: 10μm

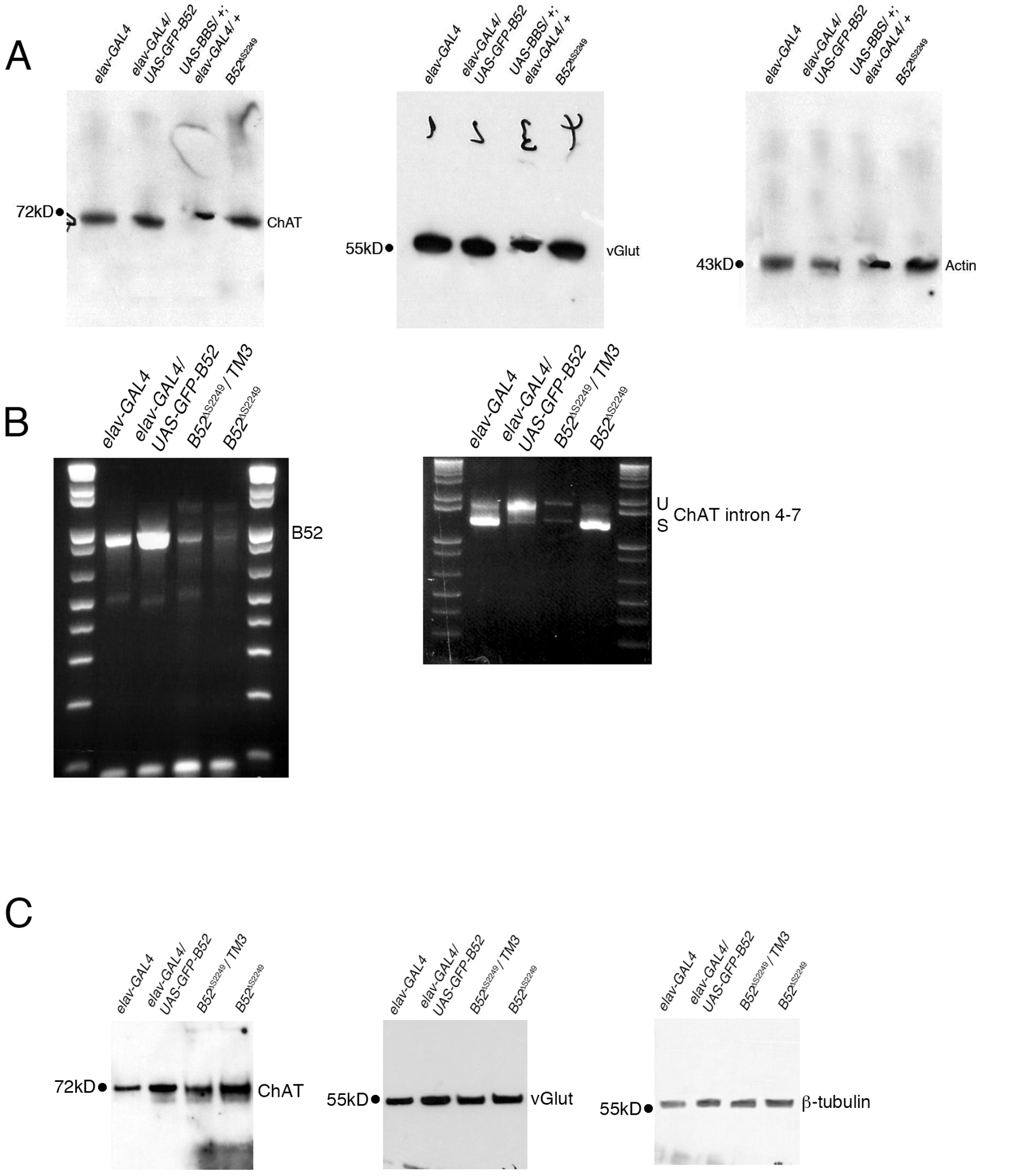

Figure S4 Uncut and unprocessed Western and RT-PCR blots

(A) Uncut image of Western blots presented in Figure 5C. (B) Uncut image of RT-PCR agarose gels presented in Figure 6A. (C) Uncut image of Western blots presented in Figure 6C. Protein weight indicated on left side of the blot in kiloDalton (kD). Type of protein or RT-PCR product indicated on right side of the blot.

Movie S1 Time-lapse recording of crawling of *B52*$^{\Delta S2249}$*/ TM3* and *B52*$^{\Delta S2249}$ mutant larvae.

Larvae were placed onto the lid of a petri dish, monitored under a dissecting microscope and recorded continuously. Arrow marks *B52*$^{\Delta S2249}$*/ TM3* sibling. Note that *B52*$^{\Delta S2249}$ homozygous mutants are not only smaller but also appear contracted.

Movie S2 Hatching movements of a control embryo *(elav::GAL4)*.

Movie covers 2h with a frame taken every 5sec. Only longer bilateral muscle contractions, which move over the embryo in peristaltic waves are apparent.

Movie S3 Hatching movements of a B52 functionally depleted embryo *(UAS-BBS/+; elav-GAL4/ +)*.

Movie covers 2h with a frame taken every 5sec. In addition to longer peristaltic waves of muscle contractions, unilateral twitches (arrows) occur.
